# LRRK 2 gene mutations in the pathophysiology of the ROCO domain and therapeutic targets for Parkinson’s disease: a review

**DOI:** 10.1186/s12929-018-0454-0

**Published:** 2018-06-14

**Authors:** Meng-Ling Chen, Ruey-Meei Wu

**Affiliations:** 1 Department of Life Science, National Taiwan University, No. 1, Sec. 4, Roosevelt Road, Da-an Dist, Taipei City, 10617 Taiwan; 2 Department of Neurology, College of Medicine, National Taiwan University Hospital, National Taiwan University, No. 7, Chung-Shan South Road, Zhongzheng Dist, Taipei City, 10002 Taiwan

**Keywords:** Parkinson’s disease, LRRK2, ROCO domain, Signaling pathway, GTPase activity

## Abstract

Parkinson’s disease (PD) is the most common movement disorder and manifests as resting tremor, rigidity, bradykinesia, and postural instability. Pathologically, PD is characterized by selective loss of dopaminergic neurons in the substantia nigra and the formation of intracellular inclusions containing α-synuclein and ubiquitin called Lewy bodies. Consequently, a remarkable deficiency of dopamine in the striatum causes progressive disability of motor function. The etiology of PD remains uncertain. Genetic variability in leucine-rich repeat kinase 2 (*LRRK2*) is the most common genetic cause of sporadic and familial PD. *LRRK2* encodes a large protein containing three catalytic and four protein-protein interaction domains. Patients with *LRRK2* mutations exhibit a clinical and pathological phenotype indistinguishable from sporadic PD. Recent studies have shown that pathological mutations of *LRRK2* can reduce the rate of guanosine triphosphate (GTP) hydrolysis, increase kinase activity and GTP binding activity, and subsequently cause cell death. The process of cell death involves several signaling pathways, including the autophagic–lysosomal pathway, intracellular trafficking, mitochondrial dysfunction, and the ubiquitin–proteasome system. This review summarizes the cellular function and pathophysiology of *LRRK2* ROCO domain mutations in PD and the perspective of therapeutic approaches.

## Background

**Fig. 1:**
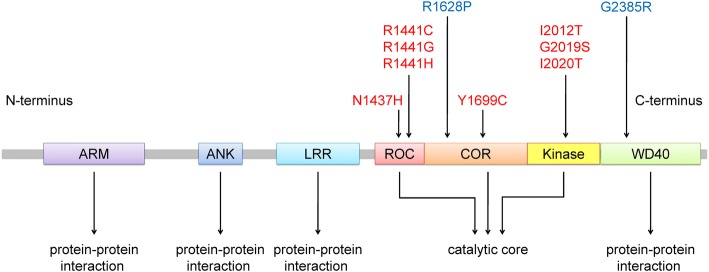
Structure of the functional domain of the LRRK2 protein and the pathogenic mutations associated with PD. ARM, armadillo; ANK, ankyrin repeat; LRR, leucine-rich repeat; ROC, Ras of complex proteins: GTPase; COR, C-terminal of ROC; WD40, WD-40 domain. Potential pathogenic mutations are shown in red, and risk polymorphisms in Asian populations are shown in blue

Parkinson’s disease (PD) is the second most common neurodegenerative disorder, affecting 1–2% of the population over 65 years of age and presents with progressive motor disability [1]. Non-motor symptoms, such as hyposmia, constipation, sleep disorder, or depression, may precede the occurrence of motor symptoms [2–6]. Dementia and psychiatric symptoms are often found at the advanced stage of disease, which further exacerbates the clinical disability and quality of life, and intensifies the burden of caregivers and social economics [7–9] The pathological hallmark of PD is the identification of intra-neuronal inclusions, or Lewy bodies, in many of the surviving cells of all affected brain regions and loss of dopaminergic neurons within the substantia nigra pars compacta. Lewy bodies are spherical, eosinophilic, cytoplasmic aggregates of a fibrillary nature that are composed of a variety of proteins, including α-synuclein (ASYN), ubiquitin, and neurofilaments [10, 11]. PD can be classified as familial or sporadic based on the heritability of its genetic origin. The proportion of families with inherited PD is between 10 and 15% [12]. To date, at least 23 loci and 19 disease-causing genes for parkinsonism have been found, but many more genetic risk loci and variants for the sporadic phenotype have been identified in various association studies [13]. LRRK2-associated PD is remarkable due to the mutations in the *LRRK2*, which are the most frequent genetic cause associated with autosomal dominant PD (ADPD) [14]. *LRRK2* is a large gene spanning a genomic region of 144 kb with 51 exons and encodes a multidomain protein consisting of 2527 amino acids. This protein belongs to the ROCO (ROC and COR domain) protein family and is made up of five characteristic functional domains: leucine-rich repeats (LRR domain), Rasp of complex proteins (ROC domain), C-terminal of Roc (COR domain), mitogen activated protein kinase kinase kinase (MAPKKK domain), and the WD40 domain. More than 80 missense mutations have been described in *LRRK2*, but only eight pathogenic mutations (Fig. 1) [15, 16]. In this review, we summarize the cellular function and pathophysiology of the LRRK2 ROCO domain in the genetics of PD and therapeutic approaches targeting this domain for PD.

### Genetic aspects of *LRRK2*-associated PD

A novel locus on chromosome 12p11.2-q13.1, *PARK8*, was first identified in 2002 in a family in Sagamihara, Japan, consisting of 31 individuals in four generations with autosomal dominant parkinsonism [17]. In 2004, Zimprich et al. identified pathogenic mutations in a novel gene in the *PARK8* locus, *LRRK2*. R1441C and Y1699C were identified in a western Nebraska family and a German–Canadian family, respectively [18]. Another study reported pathogenic mutations (R1441G) associated with the *PARK8* locus in five families from England and Spain [19]. In 2005, several simultaneous studies reported two additional pathological mutations (R1441H and G2019S) associated with both familial and sporadic PD [14, 20–23]. In the same year, a novel mutation (I2012T) was identified in a Taiwanese family [21]. In 2010, another novel pathogenic mutation (N1437H) was reported in a Norwegian family [24].

More than 80 mutations in *LRRK2* have been reported. However, only eight mutations in *LRRK2*, including N1437H, R1441 G /H/ C, Y1699C, I2012T, G2019S, and I2020T, have been proven to cause PD (Fig. 1) [16, 25, 26]. Among these pathogenic mutations, G2019S is the most common; the second most common is the R1441 “hotspot” amino acid codon residues of glycine (G), histidine (H), and cysteine (C) individually.

The various LRRK2 mutations occur with different incidence and prevalence rates in different ethnic populations. The global prevalence of the G2019S mutation has been estimated to be 1% in patients with sporadic PD and 4% in familial cases with ADPD [27]. Generally, the G2019S mutation is more frequent in the North African population (30–42% in familial and 30–34% in sporadic PD cases), in the Ashkenazi Jewish population (28% in familial and 10% in sporadic PD cases), and in the European and North American population (6% in familial and 3% in sporadic PD cases) [27–32]. However, this mutation has rarely been found (< 0.1%) among Asian populations [22, 33, 34]. The worldwide frequency of the remaining seven pathogenic mutations appears to be low, with the exception of R1441G, which is most prevalent in the Basque region, accounting for 16.4–46% of familial PD cases and 1.7–4% of sporadic PD cases in Spain [35–37]. R1441H occurs in a diverse range of ethnic groups, including Taiwanese, North American (United States), Portuguese, Greek, and Mexican [22, 23, 26, 38–43]. Conversely, the I2012T mutation appears to be geographically restricted to Taiwan [16, 21, 44].

Some LRRK2 substitutions have been associated with the risk of sporadic PD. The most common variants in Asian populations are G2385R and R1628P [45–47]. However, a few studies have reported that R1628P is not associated with PD risk in Taiwan and mainland China [48, 49].

### LRRK2 multidomain structure

**Fig. 2:**
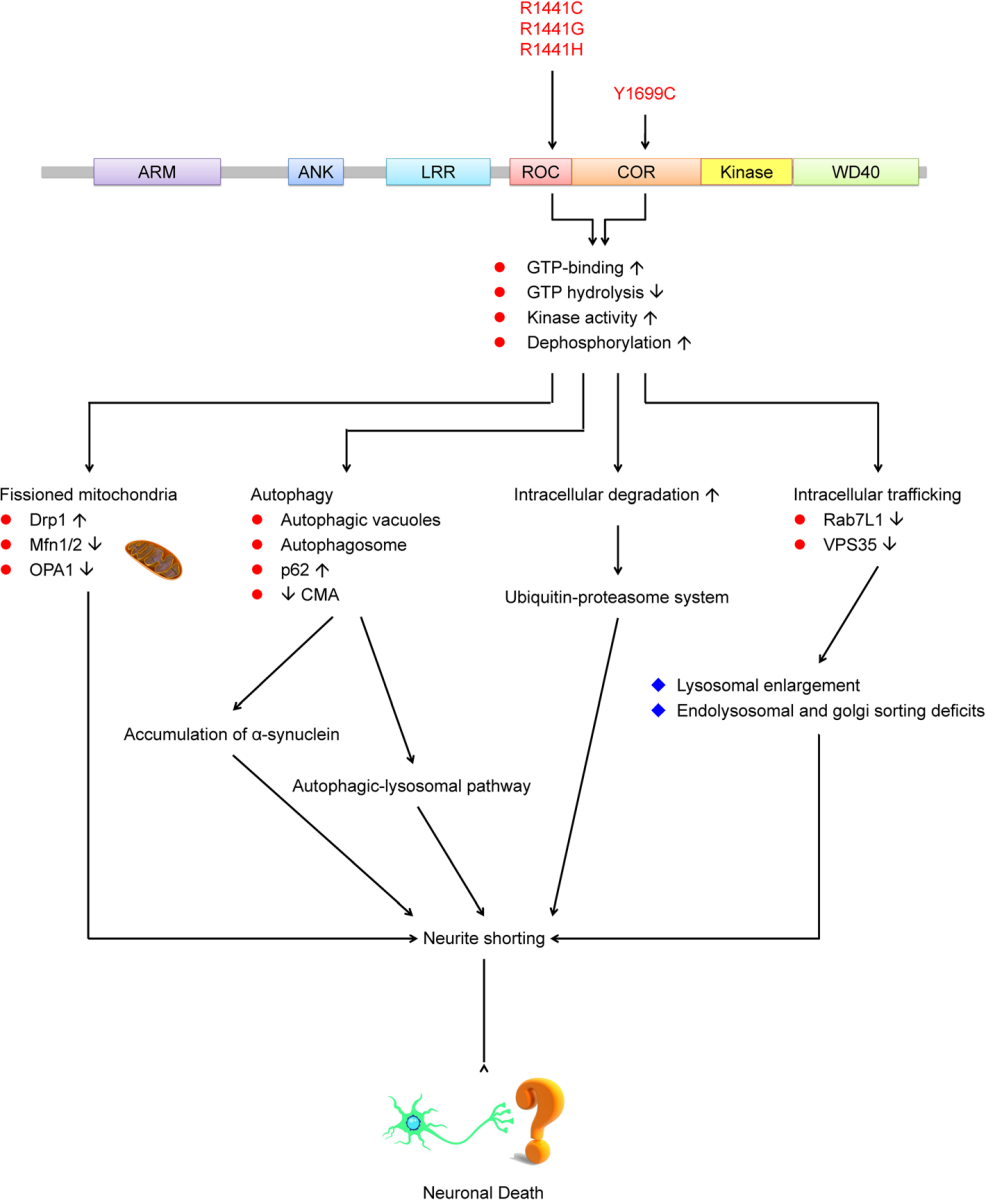
Summary of the putative mechanisms of mutations in the LRRK2 ROCO domain. Data indicate that mutations in the LRRK2 ROC domain through the alteration of kinase and/or GTPase activity can affect mitochondrial function, the ubiquitin-proteasome system, the autophagy-lysosomal pathway, and the trafficking of vesicles and proteins

*LRRK2* (*PARK8*) first received attention for its strong relationship with several *Dictyostelium discoideum* genes affecting cytokinesis, cell polarity, and chemotaxis [50–53]. Bosgraaf and Van Haastert identified a novel group in the Ras/GTPase superfamily, called Roc, which included all of the aforementioned *D. discoideum* genes plus genes found in prokaryotes, plants, and animals. One of the animal genes, called “human Roco2”, corresponds to *LRRK2* [54]. Mutations in *LRRK2* were later found to cause ADPD [18]. Mammalian LRRK2 is a 2527-residue protein with a catalytic core domain, kinase domain, and a number of putative protein–protein interaction domains (Fig. 1). The catalytic core domain consists of a Ras GTPase-like domain, termed ROC, which is followed by the COR domain immediately before the kinase domain. The ROC domain resembles typical Ras-related small GTPases, which bind and hydrolyze guanosine triphosphate (GTP) [55]. The kinase domain has similarity to MAPKKKs belonging to the serine/threonine and tyrosine kinase superfamily, which play a central role in mediating cellular stress events. The protein–protein interaction domains include the N-terminal armadillo (ARM) domain, ankyrin (ANK) repeats, 13 LRRs, and 7 C-terminal WD40 repeats [54, 56, 57]. These domains in LRRK2 may interact with or implement biochemical reactions and participate in different cellular signaling pathways. LRR-containing proteins are involved in many biologically vital processes, such as hormone–receptor interactions, enzyme inhibition, regulation of gene expression, apoptosis, and regulation of cytoskeletal dynamics, cell adhesion, cellular trafficking, neuronal differentiation, and neural development [58, 59]. WD40 is also a conserved protein–protein interaction domain involved in a broad range of cellular functions, including signal transduction, mRNA processing, transcription, cytoskeletal assembly, and mitochondrial fission [60]. The overall structure suggests that LRRK2 acts as a scaffold for other proteins and can integrate and modify multiple signaling pathways (Fig. 2).

### Role of the ROC domain in LRRK2 activity and function

LRRK2 has a dual role with both kinase and GTPase activity because it contains two distinct enzymatic domains: the kinase domain and the ROC–COR GTPase domain. Regulation of the LRRK2 kinase activity depends on the ROC domain forming a dimer via the COR domain, which possibly acts as a molecular hinge.

#### Kinase activity of LRRK2

LRRK2 kinase activity has been monitored through its autophosphorylation and its phosphorylation of a number of exogenous substrates in in vitro kinase assays using the full-length recombinant protein [61–64]. The kinase activity is induced by the formation of an LRRK2 dimer [65, 66]. Notably, LRRK2 purified from FLAG-LRRK2 BAC transgenic mouse brains exhibits enhanced kinase activity compared to the lung tissue or cultured HEK293 cells transfected with LRRK2 [67]. The G2019S and I2020T kinase domain mutations have been reported to phosphorylate mitogen-activated protein kinase kinases, including MKK3–4, − 6, and − 7, and this phosphorylation activity corresponds with LRRK2 autophosphorylation [68]. All relevant studies have consistently shown that the G2019S mutation significantly increases kinase activity [57]. Conversely, studies have reported that the I2020T mutation causes a moderate but significant increase in kinase activity, whereas other studies have reported no effect, or even a slight decrease [61, 63, 69–72].

In vitro studies involving various cell lines and primary neuronal cultures have shown that the kinase activity of LRRK2 contributes to the toxic effects of PD-associated protein variants. PD-associated protein variants include I1371V, R1441C, R1441G, Y1699C, G2019S, I2012T, and I2020T, which increase the kinase activity and cause neuronal cell death [63, 64, 73, 74]. According to these findings, overexpression of wild-type or mutant LRRK2 (R1441C, Y1699C, G2019S, and I2020T) causes both cell toxicity and cell death. When cells were treated with hydrogen peroxide, mutant LRRK2 caused remarkably more toxicity than the wild-type protein [63, 74, 75]. In contrast, expression of kinase-dead mutant LRRK2 was innocuous [63, 64, 73, 74]. These findings suggest that reactive oxidative stress contributes to cell viability and survival.

Skibinski et al. showed that LRRK2 kinase inhibitors and kinase-dead G2019S/D1994A double mutants reduce G2019S LRRK2–mediated toxicity in a well-established cell line over-expressing G2019S, which increased LRRK2 kinase activity in regards to both autophosphorylation and phosphorylation of exogenous kinase substrates [76]. This result provides compelling evidence that LRRK2 toxicity is kinase dependent. Recently, Ray et al. found that I2020T increases kinase activity through stabilization of the active-state conformation and increases the rate of phosphoryl transfer [77]. Martin et al. observed enhanced phosphorylation of the LRRK2 substrate Rps15 in G2019S and I2020T mutant cells, but not in R1441C/G mutant cells [78].

#### GTPase activity of LRRK2

The ROC domain of LRRK2 has been identified as a genuine and functional GTPase that can bind and intrinsically hydrolyze GTP in vitro [55, 63, 64, 67, 79, 80]. Several in vitro studies have shown that free GTP, guanosine diphosphate (GDP), and the non-hydrolyzable analog GTPγS compete for the GTP binding site, demonstrating that LRRK2 is an authentic GTPase [63, 64, 79]. However, in vitro studies have indicated that LRRK2 exhibits a poor ability to convert GTP to GDP. This may be related to the deficiency of suitable guanine nucleotide exchange factors or GTPase-accelerating proteins (GAPs) required for valid hydrolysis activity [55, 63, 67, 79, 80]. Recent in vitro studies indicate that rho guanine nucleotide exchange factor 7 interacts with LRRK2 to affect GTP hydrolysis activity, whereas the guanine exchange nuclear factor GAP reduces GTP hydrolysis and markedly increases LRRK2 kinase activity [81, 82]. The GTP-binding-deficient mutant T1348 N has reduced kinase activity, suggesting that GTP binding is essential to the protein kinase activity of LRRK2 [79]. Notably, FLAG-LRRK2 purified from transgenic mouse brains exhibits both GTP binding and hydrolysis activity [67]. The effect of ROCO mutations on GTPase activity in an animal model requires further elucidation.

Several studies have indicated that two pathological mutations, R1441C and R1441G, not only reduce the rate of GTP hydrolysis, but also alter the binding of GTP compared to wild-type LRRK2 [55, 67, 80]. These results may be attributable to elevated steady-state levels of GTP-bound LRRK2 [63]. Similar studies have shown that the Y1699C variant also increases the binding of GTP and reduces GTPase activity [63, 83]. In contrast with R1441C/G and Y1699C mutations, G2019S, I2012T, and I2020T mutations do not alter GTP binding [63]. However, increased GTP-bound LRRK2 in a steady state may be indicative of impaired GTP hydrolysis. Studies have suggested that a decreased rate of GTP-to-GDP conversion keeps the variants containing R1441C/G in a GTP-bound, and thus active, state [55, 67, 80].

#### LRRK2 and related signaling pathways

LRRK2 consists of seven functional domains, including catalytic and protein-protein interaction domains. Therefore, it is paramount to understand the physiological function and signaling pathways related to this protein. LRRK2 is expressed not only in human tissues (encompassing midbrain, distal ileum, spleen, and mesenteric lymph nodes), but also in peripheral blood mononuclear cells (PBMCs), including monocytes, T-cells, and B-cells, in neurologically healthy subjects [84, 85]. Patients with Crohn’s disease and chronic inflammatory bowel disease have increased expression of LRRK2 mRNA in the inflammatory area [85]. Furthermore, in the cultured bone marrow of LRRK2 R1441C mice, autophagy is reduced after exposure to several microbial structures [84]. These studies suggest that LRRK2 is involved in the immune system. Potential LRRK2-interacting proteins have been identified through immunoprecipitation and mass spectrometry assays. According to their physiological functions, the majority of these proteins can be subdivided into four groups: chaperone-mediated responses, cytoskeleton and trafficking, phosphorylation and kinase activity, and others [86]. Therefore, over the past decade, numerous signaling pathways, including the autophagic–lysosomal pathway, intracellular trafficking, and mitochondrial dysfunction, have been shown to be associated with LRRK2 in various cell and animal models. Recently, a study reported the ubiquitination and degradation of a significant fraction of LRRK2 via dephosphorylation of Ser935 after inhibition of LRRK2 kinase activity [87].

##### Mitochondrial dysfunction

Wild-type LRRK2 has multiple regulatory roles in mitochondrial fusion and fission, as various studies have shown that it interacts with some key regulators of mitochondrial fission and fusion, and colocalization studies have indicated that it exists in both the cytosol and on mitochondrial membranes [88, 89]. In studies on murine primary neurons and human neuroblastoma, the interaction between endogenous LRRK2 and the fission regulator dynamin-related protein 1 (Drp1) increased Drp1 phosphorylation and mitochondrial fission [89, 90]. This LRRK2- and Drp1-dependent mitochondrial fragmentation is enhanced by overexpression of wild-type and R1441C LRRK2 but can be reversed by inhibiting Drp1 or increasing fusion [89, 91]. Furthermore, kinase-dead or GTP-binding-deficient LRRK2 exhibits greatly reduced Drp1 interactions [92]. Studies have shown that the phosphorylation of Drp1 at S616 causes fission. In human studies, increased S616 phosphorylation has been observed in patients with sporadic PD [93, 94]. LRRK2 also interacts with the mitochondrial fusion regulators Mfn1/2 and OPA1, which modulates their activities. Furthermore, a decreased level of mature OPA1 has been noted in patients with PD carrying the R1441C mutation [89]. These findings suggest that mutation of LRRK2 in the ROCO domain decreases mitochondrial fusion and increases fission. Therefore, regulation of LRRK2 kinase activity may be a critical factor in mitochondrial fission and fusion in sporadic PD. overexpression of wild-type and/or mutant LRRK2 induces various effects on mitochondrial and cellular health [26]. These effects include a reduction of adenosine triphosphate and increased mitochondrial fragmentation, which produces more ROS, resulting in increased cell sensitivity. Furthermore, increased oxidative stress and cell death and impaired neuronal differentiation have been noted in iPSC-derived dopamine neurons from R1441C mutation carriers [95].

##### Autophagic–lysosomal pathway

Autophagy is a highly conserved and regulated process that maintains cellular homeostasis and protects cells against starvation and microbial invasion via the lysosomal pathway to control the degradation of proteins, organelles, structures, and aggregates [96]. Three types of autophagy are currently known in mammalian cells: Microautophagy, chaperone-mediated autophagy (CMA), and macroautophagy [97]. Macroautophagy, which is usually referred to simply as autophagy, is the strategy commonly used for bulk degradation of cytoplasmic proteins and organelles, including dysfunctional mitochondria, the selective degradation of which is sometimes referred to as mitophagy. Microautophagy is a much simpler process and occurs when lysosomes engulf cytosolic components directly through membrane involution. CMA incorporates cytosolic proteins brought to the lysosome membrane by chaperones.

Perinuclear lysosomal localization could promote autophagy through colocalization with autophagosomes, as well as reduced mTOR signaling [98], suggesting a possible role of LRRK2 in autophagy. Several studies have analyzed the role of LRRK2 in autophagy using different approaches and models. Pathogenic LRRK2 variants may affect either macroautophagy or CMA, though there is a lack of consensus on their central effects on the autophagic-lysosomal pathway. LRRK2 associates with autophagic vesicles and multivesicular bodies, both of which belong to abnormal structures in R1441C-expressing cells [99] and in human brain and cultured cells. The disruption of autophagy in midbrain dopamine neurons through Atg7 conditional knockout leads to eventual neuronal death and locomotor deficits in mice [100, 101], highlighting a possible connection between aberrant autophagy and neurodegeneration. Conversely, G2019S leads to augmented autophagy in various cells, possibly via mechanisms including mitochondrial fragmentation with elevated Drp1 phosphorylation [89–91, 102].

The LRRK2 ROCO mutant has been found to be a degradative substrate for CMA. R1441C and wild-type overexpression reduce the capacity for CMA, as indicated by the accumulation of ASYN and misfolded proteins, which is generally seen in PD. This may be, at least partially, the result of LRRK2-mediated alteration of cellular proteolytic pathways [103]. Although pathogenic LRRK2 variants are poorly degraded through CMA, LRRK2 degradation occurs through both the ubiquitin–proteasome system and CMA, and LRRK2 impedes the uptake of other CMA substrates, including ASYN. This may be a mechanism of its toxicity [103].

However, whether LRRK2 plays a positive or negative regulatory role in the control of macroautophagy and whether it functions in the initiation step or the clearance step is still controversial. This open debate has been highlighted by the study of LRRK2-knockout animals. Though the brains of LRRK2-knockout mice in one study did not exhibit the pathological hallmarks of PD, a biphasic alteration in macroautophagy was observed in the kidneys, with enhanced autophagy at young ages and reduced autophagy at old ages [104]. An impaired response to starvation-induced macroautophagy was evident across G2019S, Y1699C, and R1441G mutations [105]. However, the details of the molecular mechanism remain vague.

Mutations in the GTPase domain (e.g., R1441C) cause an accumulation of autophagic vacuoles, with increased levels of p62 as a marker of autophagy in HEK-293 cells [99]. In mouse models, LRRK2 knockout causes striking age-dependent accumulation and aggregation of ASYN and ubiquitinated proteins in the kidney. The autophagy–lysosomal pathway is also impaired in the absence of LRRK2, involving lipofuscin granule accumulation and altered levels of LC3-II and p62 [104]. An investigation of the regulation of the tissue specificity of LRRK2 expression by autophagy showed the age-dependent accumulation of autophagic vacuoles in the cortex and striatum of R1441C and G2019S transgenic mice, suggesting that LRRK2 expression is only regulated by autophagy in neuronal somas and axial processes from the cortex and striatum [106].

##### Intracellular trafficking

Several studies suggest that LRRK2 plays a role in vesicle trafficking by interacting mainly with trafficking proteins, such as endophilin A, Rab7, Rab7L1, and members of the dynamin GTPase superfamily. Evidence of LRRK2 paralog LRRK1-mediated EGFR endocytosis [107] supports the role of LRRKs in vesicle formation and transportation. *Drosophila* studies have indicated prominent potential roles of LRRK2 in multiple aspects of vesicle trafficking, including synaptic vesicle recycling, retromer trafficking, and lysosomal positioning. LRRK2 may be enriched at the Golgi complex [88, 108]. Genetic interaction studies have indicated the participation of LRRK2 in the retromer complex, which mediates retrograde transport of proteins, such as acid hydrolase receptors, from endosomes to the trans-Golgi network [109]. In cultured rat neurons, overexpression of VPS35, a component of the retromer complex with mutations identified in familial PD [109], rescues cells from both lysosomal enlargement and endolysosomal and Golgi sorting deficits triggered by R1441C expression. However, expression of the PD-linked mutant VPS35 fails to reverse these defects. Consistent with LRRK2-mediated retromer dysfunction, overexpression of Rab7L1, another retromer component implicated in PD in primary rat neurons overexpressing R1441C, can reverse the shortening phenotype. Rab7L1 localizes to the trans-Golgi network and has been suggested to be part of a LRRK2 complex that operatively promotes autophagy of the trans-Golgi network [110]. The fly LRRK2 homolog dLRRK associates with membranes of late endosomes and lysosomes and physically interacts with Rab7, which promotes perinuclear clustering of lysosomes during starvation [111]. The evidence indicates that dLRRK negatively regulates lysosomal transport towards nuclei.

##### Ubiquitin–proteasome system

**Fig. 3:**
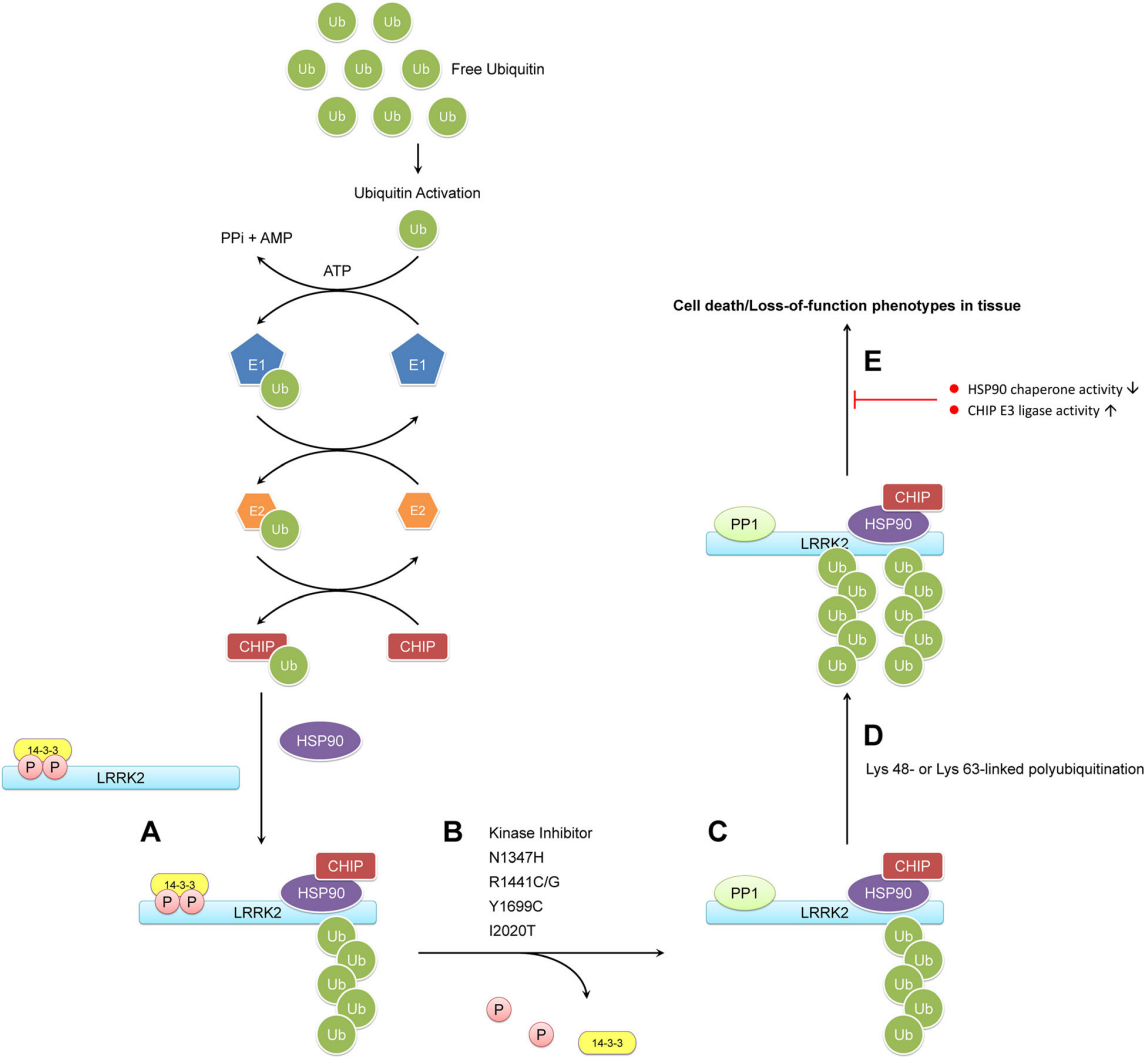
Scheme summarizing the LRRK2 putative mechanism in ubiquitination. **a** LRRK2 probably exists in a basal ubiquitinated (Ub) state regulated by CHIP and HSP90 to maintain LRRK2 protein stability. LRRK2 also occurs in a phosphorylated (P) state that is bound to 14–3-3 proteins. **b** In the presence of kinase inhibition or pathogenic PD-related mutations, including N1437H, R1441C, Y1699C, and I2020T, protein phosphatase 1 (PP1) is recruited to LRRK2, causing dephosphorylation and loss of 14–3-3 binding. **c** Dephosphorylation of LRRK2 promotes the addition of ubiquitin to LRRK2 through Lys48 or Lys 63-linked polyubiquitin chains. **d** This leads to degradation or potentially differential signaling of LRRK2 via ubiquitin linkages. **e** Increasing CHIP E3 ligase activity and blocking HSP90 chaperone activity can prevent the deleterious effects of LRRK2 and enhance cell viability

LRRK2 protein stability is regulated by carboxyl terminus of HSP70-interacting protein (CHIP), an E3 ubiquitin ligase, whereas CHIP and HSP90 levels are critical determinants of LRRK2 toxicity [112–114]. The destabilization of LRRK2 by CHIP is due to CHIP-mediated ubiquitination and proteasome-dependent degradation [113]. CHIP interacts with and ubiqiutinates LRRK2, leading to the latter’s proteasomal degradation through an HSP90 chaperone–containing complex [112]. A summary of the LRRK2 putative mechanism in ubiquitination is illustrated in Fig. 3.

Zhao et al. found that GNE1023, an inhibitor of LRRK2 kinase activity, causes dephosphorylation of LRRK2 at Ser935, which is localized between the ANK and LRR domains, in HEK293 cells over-expressing LRRK2 [87]. GNE1023 also induced LRRK2 degradation in cell and mouse models through Lys48 and Lys63 ubiquitin linkages. In human epithelial cells transfected with wild-type LRRK2, treatment with GNE1023 alone or in the presence of mutant Lys48 or Lys63 linkages drove LRRK2 to accumulate in the cytoplasm and form filamentous skein-like structures. Furthermore, GNE1023 plus either ubiquitin-linkage mutant appears to strengthen the accumulation of LRRK2 [87].

The 14–3-3 proteins play various physiological roles and interact with a multitude of substrate proteins [115, 116]. Several studies have shown that 14–3-3 binding could regulate cytoplasmic distribution, protect from dephosphorylation, and be involved in extracellular secretion of LRRK2 [117–120]. Interestingly, dephosphorylation of LRRK2 at S935 increases LRRK2 ubiquitination by 14–3-3 inhibitor. The ubiquitination level was similar to GNE1023 treatment [87]. Thus, the dephosphorylation of LRRK2 at S935 is sufficient for modulating the ubiquitination and degradation of LRRK2. The pathogenic PD-related mutations, including N1347H, R1441C/G, Y1699C, and I2020T, were previously found to be more dephosphorylated than the G2019S mutant and wild-type, but new data have revealed that the basal level of ubiquitination of the G2019S mutant and wild-type is higher (Fig. 3b) [118, 121]. One study recently reported that blockage of protein phosphatase 1 (PP1) with calyculin A restores phosphorylation of the upstream kinase sites [33]. Conversely, PP1 inhibition restores phosphorylation at the upstream sites for all mutants, leading to a minimally ubiquitinated LRRK2 species (Fig. 3c).

Defining the ubiquitination linkage types of LRRK2 under various pathogenic conditions and determining tissue- or cell-population-specific differences will be vital in future studies [87]. Studying the phosphorylation, ubiquitination, and degradation cycle in physiological LRRK2 animal models will also be crucial. How this cycle differs among LRRK2 inhibitor types, such as kinase and general inhibitor, in G2019S and R1441C knock-in mice is of interest [122–124].

### Therapeutic approaches targeting LRRK2 GTPase activity and GTP binding

Early therapeutic approaches targeting LRRK2 focused on its kinase activity, and several published reports address kinase domain inhibitors for LRRK2. However, increasingly more studies have shown that the GTP domain plays vital roles in LRRK2 biological functions. One study showed that the R1441H mutant causes a 2-fold increase in GTP binding activity and kinase activity compared to wild-type LRRK2 [125]. These findings suggest that alteration of the LRRK2-GTP domain or GTP binding is a novel effective therapeutic target for PD.

Li et al. discovered that two compounds (**68** and **70**) that reduce GTP binding and inhibit kinase activity in vitro and in cultured cells can attenuate neuronal degeneration in cells [126]. Compound **68** also reduced GTP-binding activity and kinase activity in the brain after intraperitoneal injection in a LRRK2-based lipopolysaccharide-induced pre-inflammatory mouse model [126]. However, compound **68** has low blood–brain barrier permeability. To solve this problem, FX2149, a novel analog of **68**, was developed. This compound has improved in vivo efficacy and retains the inhibition of GTP binding to LRRK2 [127]. Furthermore, reducing GTP-binding activity with compound **68** and FX2149 attenuated the impairment of mitochondrial and lysosomal transport in cells expressing R1441C [128].

## Conclusion

The ROC domain of LRRK2 has been identified as a functional GTPase which regulates the LRRK2 kinase activity depending on the formation of a dimer via COR domain. PD-associated protein variants in ROCO and Kinase domains including I1371V, R1441C, R1441G, Y1699C and G2019S, I2012T, and I2020T, which increase the kinase activity and cause neuronal cell death. Mutations in the *LRRK2* ROCO domains lead to the dysregulation of mitochondrial dynamics and abnormal changes of autophagic–lysosomal pathway, intracellular trafficking and ubiquitin–proteasome system. Therefore, the explanation of the LRRK2 ROCO domain is likely to elucidate the LRRK2 pathogenic mechanism and open venues for developing the therapies basing on the signal transduction cascades of LRRK2 for diseases arising from LRRK2 dysfunction. This review also improves our understanding of LRRK2 functions in the pathobiology of PD and identifies a potential novel strategy for treating PD.

## Abbreviations

ADPD: autosomal dominant PD
ANK: ankyrin
ARM: armadillo
ASYN: α-synuclein
CHIP: carboxyl terminus of HSP70-interacting protein
CMA: chaperone-mediated autophagy
COR: carboxyl-terminal of Ras
Drp1: dynamin-related protein 1
LRR: leucine-rich repeat region
LRRK2: leucine-rich repeat kinase 2
MAPKKK: mitogen-activated protein kinase kinase kinase
PD: Parkinson’s disease
PP1: protein phosphatase 1
ROC: Ras of complex protein
